# Magnetostructural coupling in *R*FeO_3_ (*R* = Nd, Tb, Eu and Gd)

**DOI:** 10.1038/s41598-022-13097-1

**Published:** 2022-06-11

**Authors:** R. Vilarinho, M. C. Weber, M. Guennou, A. C. Miranda, C. Dias, P. Tavares, J. Kreisel, A. Almeida, J. Agostinho Moreira

**Affiliations:** 1 IFIMUP, Departamento de Física e Astronomia, Faculdade de Ciências, Universidade do Porto, rua do Campo Alegre s/n, 4169-007 Porto, Portugal; 2 Department of Materials, ETH Zurich, Vladimir-Prelog-Weg 4, 8093 Zurich, Switzerland; 3 Institut des Molécules et Matériaux du Mans, UMR 6283 CNRS, Le Mans Université, 72085 Le Mans, France; 4 Department of Physics and Materials Science, University of Luxembourg, 41 Rue du Brill, 4422 Belvaux, Luxembourg; 5 Centro de Química, Departamento de Química, Universidade de Trás-os-Montes e Alto Douro, 5000-801 Vila Real, Portugal

**Keywords:** Condensed-matter physics, Magnetic properties and materials, Phase transitions and critical phenomena, Raman spectroscopy

## Abstract

We investigate the interplay of magnetization and lattice vibrations in rare-earth orthoferrites *R*FeO_3_, with a specific focus on non-symmetry-breaking anomalies. To do so, we study the magnetization, magnon excitations and lattice dynamics as a function of temperature in NdFeO_3_, TbFeO_3_, EuFeO_3_ and GdFeO_3_. The magnetization shows distinct temperature anomalous behavior for all investigated rare-earth orthoferrites, even in the compounds with no phase transitions occurring at those temperatures. Through spin–phonon coupling, these magnetic changes are mirrored by the FeO_6_ rotation mode for all the studied *R*FeO_3_, revealing a common magnetostructural effect associated with the octahedra rotations. The *R*^3+^ oscillation modes evidence a Fe^3+^/R^3+^ spins cross-talk for the NdFeO_3_ and TbFeO_3_ cases. Our work sheds light into the common magnetostructural coupling in rare-earth orthoferrites, and the important role of magnetic anisotropy and spin–orbit coupling strength of the *R*–Fe interactions on the spin-reorientation transition at high temperatures.

## Introduction

Rare-earth orthoferrites *R*FeO_3_ (*R* = rare-earth trivalent cation), despite being studied for decades^1–6^, attracted recent scientific interest thanks to their intriguing magnetic properties, including spin-reorientation transitions and new-found magnetically induced ferroelectricity^7–12^.

The Fe^3+^ spins order at T_N_ between 623 and 740 K, with increasing temperature for increasing rare-earth cation size^5,13^. The ordering is triggered by the condensation of the magnetic order parameter with symmetry mΓ_4_^+^. This gives rise to an A_x_F_y_G_z_-type ordering of the Fe^3+^ spin sublattice (in Bertaut notation)^14^ with the magnetic space group P*n’ma’.* The primary magnetic order is of the antiferromagnetic G-type. The canted spin structure leads to a net-magnetization along the *b*-axis^5,13^. The *R*^3+^ spin-sublattice orders below 10 K. Like the Fe^3+^ spin-sublattice, a G-type order dominates the magnetic structure of the *R*^3+^ spin-sublattice. The interaction of both G-type lattices can give rise to improper ferroelectricity, as for instance in GdFeO_3_ below 3 K^9,12,15,16^.

Between T_N_ and the temperature of *R*^3+^ spin ordering, the magnetic landscape is dominated by the crosstalk of the ordered iron sublattice and the paramagnetic rare-earth spins. On the one hand, rare-earth ions with a significant magnetocrystalline anisotropy trigger a spin reorientation transition of the magnetic iron sublattice (e.g. *R* = Nd, Sm, Er and Tb)^5^. On the other hand, the iron sublattice induces a net magnetization of the rare-earth sublattice. It was suggested that parallel or antiparallel alignment of the *R*^3+^ spin sublattice with respect to the Fe^3+^ one is steered by the octahedron tilt system, thus linking the crystal structure and magnetism^17^.

In this intermediate regime, temperature-dependent magnetic anomalies were reported. For instance, the M(T) curves of GdFeO_3_ and TbFeO_3_ exhibit s-like anomalies at around 210 and 250 K, respectively^1,6^. However, their origins and link to the crystal structure and lattice vibrations remain still unclear.

Our present work is motivated by the need to shed light on these aspects aiming for a deeper understanding of the magnetic behavior in *R*FeO_3_. Towards this objective, we scrutinize the magnetization, and Raman-active magnons and phonons across a large temperature range of a series of *R*FeO_3_: NdFeO_3_, TbFeO_3_, EuFeO_3_ and GdFeO_3_. We selected these four compounds for their different rare-earth magnetic properties and interplay between magnetic and lattice degrees of freedom, to allow the access to different magnetic interactions between the Fe^3+^ and the R^3+^ spins. For instance, both NdFeO_3_ and TbFeO_3_ exhibit spin-reorientation transitions, yet at different temperatures, and hence a significant magnetocrystalline anisotropy. On the other hand, Gd^3+^ ions present no magnetocrystalline anisotropy of spin–orbit origin (i.e. zero orbital moment, with a large spin moment) and Eu^3+^ has no magnetic moment. Therefore, this set of compounds is representative for the family of orthoferrites in general. From the comprehensive study of the temperature dependence of the magnetization and magnon wavenumbers, different types of magnetic anomalies are ascertained, which are not associated with non-symmetry-breaking. These magnetic changes are mirrored in the FeO_6_-rotation and *R*-oscillation modes, via spin–phonon coupling. Our experimental results show a common magnetostructural effect occurring in these *R*FeO_3_ associated with the FeO_6_ octahedra rotations, and a cross-talking between Fe^3+^ spins and the *R*^3+^ spins for the NdFeO_3_ and TbFeO_3_ cases that exposes the importance of magnetic anisotropy and spin–orbit coupling in triggering the spin-reorientation transition.

## Methods

High-quality ceramic pellets of *R*FeO_3_ (*R* = Nd, Eu, Gd and Tb) were processed through the urea sol–gel combustion method, sintered at 1350 °C for 60 h, quenched to room temperature. X-ray powder diffraction patterns were recorded at ambient conditions using an X’Pert Pro PANalytical diffractometer with a copper anode (1.54184 Å) in Bragg–Brentano geometry and an ultrafast X’Celerator detector with a secondary monochromator, from 10° to 95° in 2θ. Rietveld refinements of the diffraction patterns confirm the correct *Pnma* space group (see Fig. S1 of Supplemental Material). No secondary phases were detected, except in NdFeO_3_ ceramics, which show an amount of 6.8% of Nd_2_O_3_.

Low-field DC induced specific magnetization measurements were carried out using commercial superconducting quantum interference SQUID magnetometer. The magnetization was measured after zero-field (ZFC) and field cooling (FC) from 5 to 380 K under an applied magnetic field of 40 Oe, with a resolution of 1 × 10^–7^ emu.

Raman spectra were recorded using a Jobin–Yvon T64000 spectrometer and a Renishaw inVia Qontor with 514.5 nm and 532 nm linearly polarized excitation lines of Ar^+^ and diode-pumped lasers, respectively. The spectral ranges cover 100 to 800 cm^−1^ and − 600 to 600 cm^−1^. Measurements were performed at fixed temperatures from 10 to 875 K using either a closed-cycle helium cryostat or a THMS600 Linkam Stage. The effect of the laser power on the sample was previously studied and it was limited below 5 mW to prevent sample heating. The best fits of a sum of damped oscillators to the measured Raman spectra allow us to determine the wavenumbers of the phonon and magnon modes^18^.

## Experimental result s and discussion

### Magnetic properties and interactions

**Figure 1:**
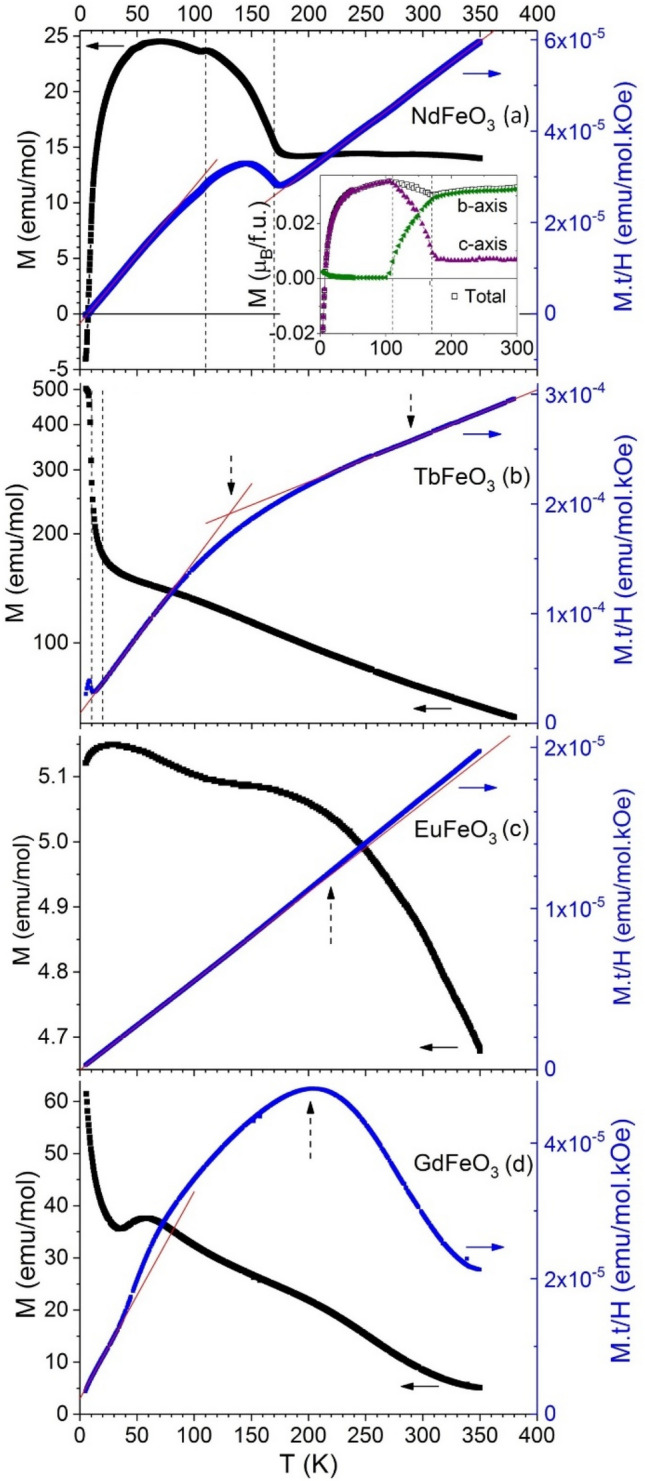
Left axes: temperature dependence of the magnetization of (**a**) NdFeO_3_ under FC conditions, and (**b**) TbFeO_3_, (**c**) EuFeO_3_ and (**d**) GdFeO_3_, under ZFC conditions, measured in heating under 40 Oe. Inset of (**a**) shows similar M(T) data for a NdFeO_3_ single crystal from Ref.^7^. Right axes: M.t/H (t = T/T_N_) versus temperature for the same compounds. Vertical dashed lines mark the phase transition temperatures following Ref.^13^, while vertical dashed arrows mark relevant anomalies.

In the first step of our study, we investigate the temperature dependence of the magnetization for NdFeO_3_ (FC), TbFeO_3_, EuFeO_3_ and GdFeO_3_ (ZFC), as displayed in Fig. 1. The vertical dashed arrows mark the temperatures of anomalies that will be important for the following sections of this work. Remarkably, the magnetization curves have little in common and the magnetization signals are dramatically different for different rare-earths. This motivates the following detailed analysis.

**NdFeO**_**3**_. The magnetization of NdFeO_3_ (Fig. 1a) is temperature independent between 350 and 175 K. With the start of the spin reorientation from *A*_*x*_*F*_*y*_*G*_*z*_ (*Pn′ma′*) to *C*_*x*_*G*_*y*_*F*_*z*_-type order (*Pn′m′a*) at 170 K, M(T) increases monotonously until a small dent at 110 K marks the end of the spin reorientation regime^19^. Down to 70 K, M(T) further increases. Hereafter, the magnetization decreases due to the gradual antiparallel ordering of the Nd^3+^ spins in the exchange field of the Fe^3+^ spins^7^. At 9 K, the magnetic contributions of the Nd^3+^ and Fe^3+^ spins compensate and the magnetization reverses for lower temperatures. The M(T) profile of the ceramic sample qualitatively agrees with the vector sum of the *b*-axis and *c*-axis components of the magnetization for single crystal measurements taken from Ref.^7^ (see inset of Fig. 1a).

**TbFeO**_**3**_. In Fig. 1b, M(T) of TbFeO_3_ increases with decreasing temperature with changes in slope for several temperature intervals. At 280 K and at 150 K, the slope of M(T) increases slightly (see Fig. S2 of Supplemental Material for a detailed view). These features were reported earlier, however, remain to be understood^6^. Below 20 K, the slope of M(T) becomes steeper with a maximum at 10 K. The sudden increase in slope at 20 K marks the onset of the spin-reorientation transition, where the net magnetic moment of the Fe^3+^ spins rotates from the *b*- to the *c*-axis. In addition, the Tb^3+^ spins order in the exchange field of the Fe^3+^ spins below 10 K, reflected by the saturation of M(T) in good agreement with the literature^10–20^.

**EuFeO**_**3**_** and GdFeO**_**3**_ do not show a spin-reorientation transition^13^. Nevertheless, the M(T) curve of both materials is characterized by anomalies with clear changes in slope. The magnetization of EuFeO_3_ increases as the temperature decreases toward its maximum at 30 K, presenting a broad anomaly around 200 K. Also a broad maximum of M(T) around 200 K was reported in the literature for EuFeO_3_, with the magnetization decreasing towards 0 K^21^. The temperature dependence of the magnetization of GdFeO_3_ is more complex, exhibiting strong variations below 100 K. For T < 36 K, the magnetization increases due to the paramagnetic response of the Gd^3+^ sublattice^22^. Qualitatively, the M(T) curve of GdFeO_3_ agrees with the results reported in the literature^1,21^. The existence of magnetic changes occurring at 260 K is also ascertained by the anomalous temperature dependence of the coercive field at 200 K^23^.

Among the four studied compounds and in the 5 to 350 K range, TbFeO_3_ presents the highest maximum magnetization value (around 500 emu/mol), while EuFeO_3_ presents the smallest one (around 0.5 emu/mol, 3 orders of magnitude smaller than TbFeO_3_). GdFeO_3_ and NdFeO_3_ are in between, with GdFeO_3_ presenting a larger magnetization below 150 K. Due to the van Vleck character of Eu^3+^, its spins are expected to contribute little to the total magnetization contrary to the other rare-earth cations^24^. Thus, the magnetization of EuFeO_3_ arises dominantly from the Fe^3+^ spin sublattice, due to the canting of the Fe^3+^ spins, which is found in literature to be 8.0 mrad^4^. Despite the similar canting angles of NdFeO_3_, GdFeO_3_ and TbFeO_3_, these compounds show a substantially larger magnetization than EuFeO_3_^4^. Therefore, we conclude that the contribution of the Nd^3+^, Gd^3+^, and Tb^3+^ paramagnetic momenta or their interaction with Fe^3+^ add a significant contribution for the overall magnetization. In the case of TbFeO_3_ and GdFeO_3_, the *R*^3+^ spins align parallelly to the net magnetization of the Fe^3+^ spins, leading to an increase of the overall magnetization. The Nd^3+^ spins, in turn, align antiparallelly to the Fe^3+^ spins. Hence, the magnetization decreases and even reverses at low temperatures.

**Figure 2:**
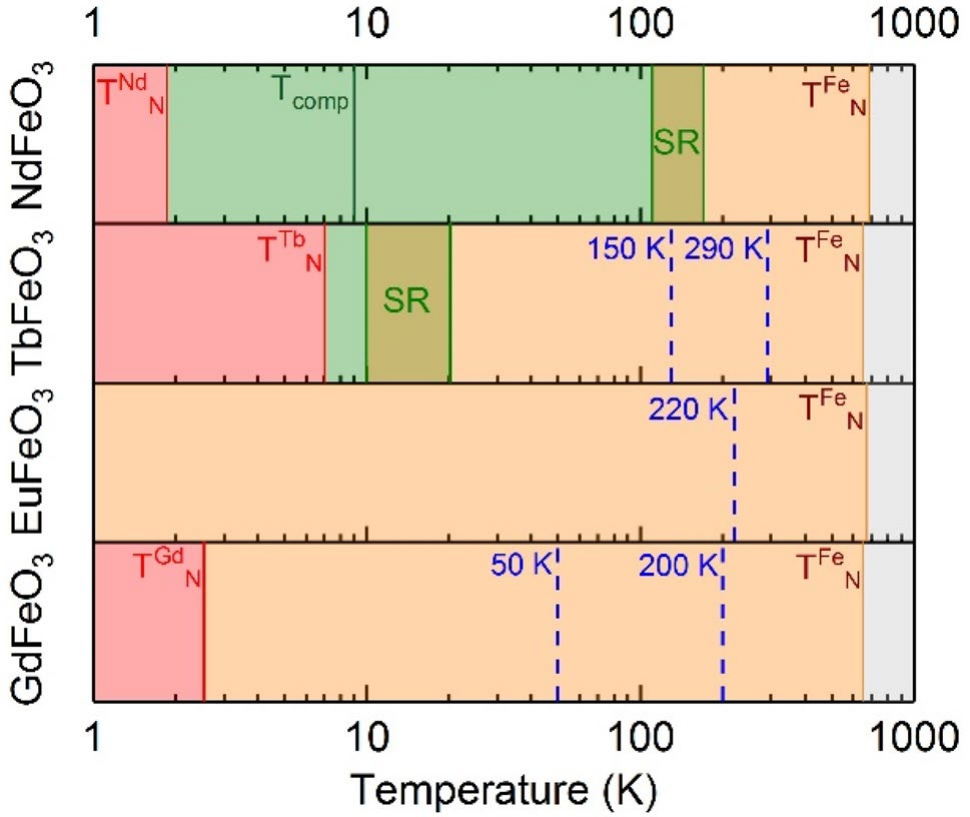
Magnetic phase sequence for *R*FeO_3_ with R = Nd, Tb, Eu and Gd. Critical temperatures of the reported phase transitions taken from Refs.^10,13,25^. Dashed lines mark relevant anomalies observed in this work on M(T) curves, not associated with phase transitions.

Figure 2 summarizes the temperatures where discernable magnetic anomalies are observed for each *R*FeO_3_. Apart from the spin-reorientations of NdFeO_3_ and TbFeO_3_, these anomalies below T_N_ do not correspond to any critical phenomena or phase transitions. The observed anomalies in the M(T) curves, which are between 3 and 4 orders of magnitude larger than the measurement resolution, indicate complex magnetic interactions in the investigated orthoferrites, triggered by the interaction between Fe^3+^ and R^3+^ spins, which we analyze in the following.

In the molecular field approximation, we can express the net magnetization resulting from the interaction of the order Fe^3+^ sublattice and the paramagnetic *R*^3+^ spins as follows^2^:

1 $$M\left(T\right)=2\alpha \sigma (T)\left[1+\frac{\langle d\rangle }{t}\right],$$

where $\alpha$ is the spin canting angle, σ(T) the Fe^3+^ sublattice moment, $\langle d\rangle$ the mean *R*–Fe interaction parameter and $t=T/{T}_{N}$. The plot of $F\left(t\right)=M\left(T\right)t/H$ versus $T$ is represented by the blue curves (right axes) of Fig. 1. Here, $H\propto \sigma (T)/\sigma (0)$ is the effective magnetic field, calculated from Mössbauer data^4^, and $\sigma (0)$ is the Fe^3+^ sublattice magnetic moment at 0 K. According to the molecular field approximation, the changes of the slope of $F\left(t\right)$, thus of $2\alpha \sigma (0)/{T}_{N}$, evidence changes of net magnetization of the iron sublattice^2^. From the intercept with $t=0$, we calculate $\langle d\rangle$ associated with the magnetic interaction strength between *R*^3+^ and Fe^3+^ spins^2^.

**NdFeO**_**3**_ presents two linear regimes above and below the spin reorientation transition. The slope in the 10 to 60 K range is about 59% steeper than above the spin reorientation. We interpret this as a consequence of the emergent magnetic interaction of Nd^3+^ and Fe^3+^ spins below the spin reorientation regime.

**TbFeO**_**3**_ also exhibits two linear regimes in the 20 to 80 K and 250 to 350 K in $F\left(t\right)$, respectively, with a slope decrease of about 70% as temperature increases, in agreement with the literature^20^. The deviation below 260 K from the high temperature linear regime can be better observed in the residuals plot obtained from subtraction of the linear fit to the data, shown Fig. S2 of the Supplemental Material.

**EuFeO**_**3**_ exhibits two different linear temperature dependences from 5 to 150 K, and 230 to 350 K, respectively, with only a minor slope change, of the order of 4%. This is in agreement with small variation of magnetization with temperature^20^.

**GdFeO**_**3**_, displays the most complex $F\left(t\right)$ behavior, with linear relations from 10 to 35 K, 100 to 150 K and 250 to 300 K and a local maximum at 200 K. Such a behavior suggests that the model does not fully reproduce the magnetic properties of GdFeO_3_ above 150 K, but this shall not invalidate the calculation of the $\langle d\rangle$ value at very low temperatures.

The calculated $\langle d\rangle$ values are presented in Table 1. For the fitting, the linear regime at low temperatures (see Table 1) was used, where the *R*–Fe magnetic interaction is strongest. The $\langle d\rangle$ values are about one order of magnitude smaller than those reported by Treves^2^, which were calculated from data available only above 100 K. For comparison with Treves, we have analyzed our data in the same temperature range, then leading to similar values. The difference is thus due to the analyzed temperature regime, and we consider that our fittings to the lower temperature range more correctly account the underlying physics.

**Table 1:** Mean *R*–Fe interaction parameter $\langle d\rangle$ calculated from the best fit of Eq. (1) to the experimental data shown in Fig. 1a–d, and the temperature interval where the fit is performed.

Compound	$\langle d\rangle \times {10}^{3}$	∆T_fit_ (K)
NdFeO_3_	− 10.24 ± 0.03	11–52
TbFeO_3_	10.3 ± 0.1	18–70
EuFeO_3_	− 0.62 ± 0.05	5–160
GdFeO_3_	11.3 ± 0.1	13–34

For NdFeO_3_, $\langle d\rangle$ is negative. This indicates antiferromagnetic interaction between Nd^3+^ and Fe^3+^ spins, in good agreement with the Nd^3+^ spin ordering antiparallel to the Fe^3+^ spins. For GdFeO_3_ and TbFeO_3_, $\langle d\rangle$ is positive. This leads to a parallel alignment of Tb^3+^/Gd^3+^ and Fe^3+^ spins and explains the increase in overall magnetization and the absence of a compensation temperature. We note, that these values are in agreement with the theoretical ones for the orientation of the rare-earth sublattice magnetization with respect to the Fe^3+^ spins sublattice^17^. Among the studied compounds, the mean interaction coupling parameter between Eu^3+^ and Fe^3+^ cations has the smallest absolute value. This indicates a comparably small interaction between both ions and can be understood by the small magnetic momentum of the Eu^3+^ cations.

It is worth to stress that, although the values of $\langle d\rangle$ are similar for both GdFeO_3_ and TbFeO_3_, the anisotropy of TbFeO_3_ is larger, such that TbFeO_3_ shows a spin-reorientation transition whereas GdFeO_3_ does not. NdFeO_3_ and TbFeO_3_ show a similar *R*–Fe interaction parameter $\langle d\rangle$, yet of different sign. Also, the spin reorientation temperatures differ significantly for both materials. These behaviors underline the complexity of magnetic interactions in rare-earth orthoferrites.

### Magnon excitations

We now investigate the magnetic changes through a temperature-dependent analysis of collective spin excitations of the Fe^3+^ spin lattice, so-called magnons. Magnons are known to be an excellent probe of subtle magnetic changes, such as the beforehand reported anomalies in *R*FeO_3_ with *R* = Y, Sm, Dy, Ho, Tm, Er and Tb^5,26–28^.

**Figure 3:**
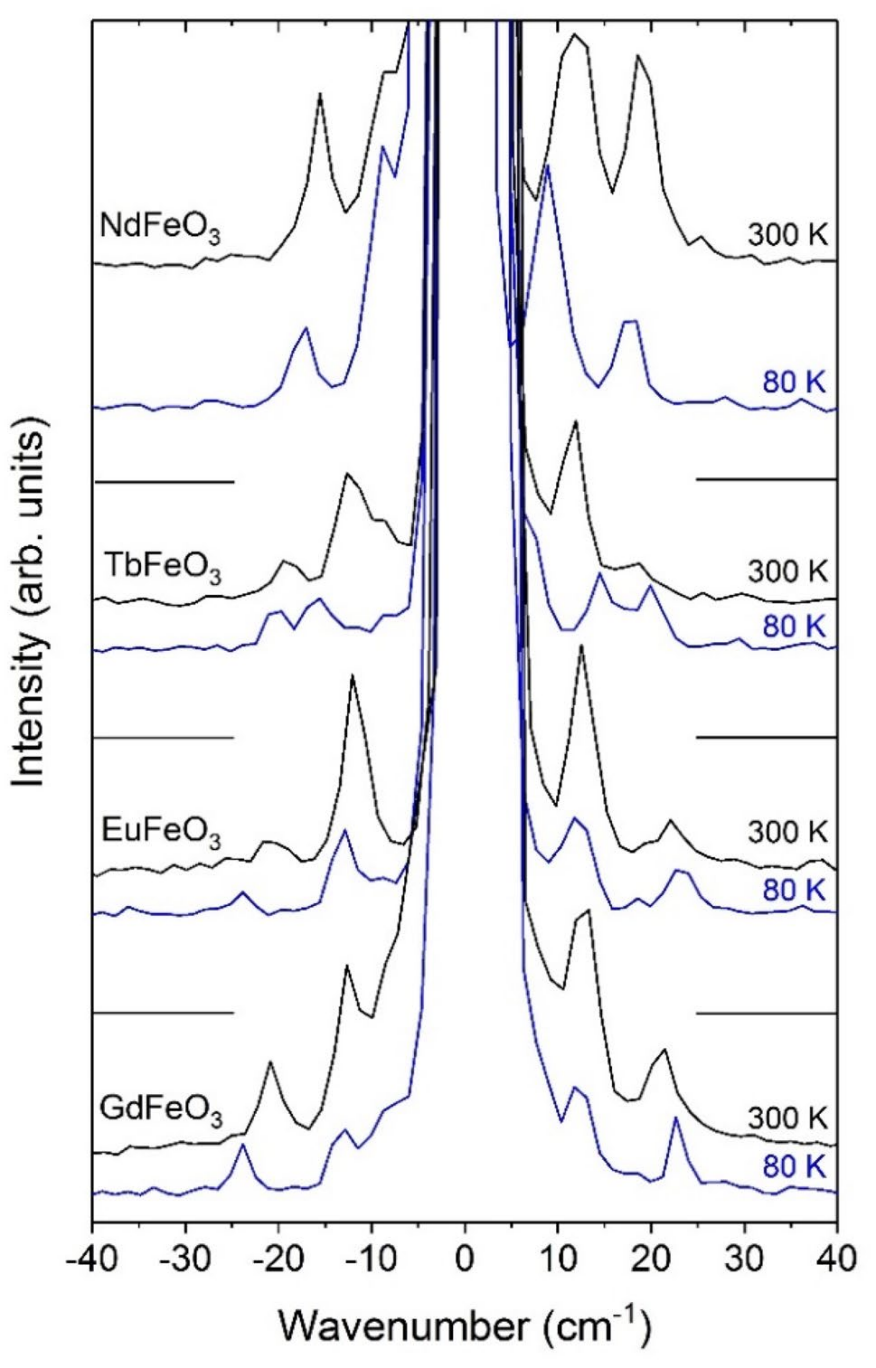
Representative unpolarized Stokes and anti-Stokes Raman spectra of *R*FeO_3_ (*R* = Nd, Eu, Gd and Tb), in the −40 to 40 cm^−1^ spectral range, recorded at 80 and 300 K.

Figure 3 shows the unpolarized Stokes and anti-Stokes Raman spectra of NdFeO_3_, TbFeO_3_, EuFeO_3_ and GdFeO_3_ recorded at 80 and 300 K. Two magnon modes are observed, in good agreement with earlier experiments on other *R*FeO_3_^26,27,29^. According to literature, the lower wavenumber mode (M1) is assigned to the ferromagnetic magnon and the higher wavenumber mode (M2) to the antiferromagnetic magnon of the Fe^3+^ spin-sublattice^26,27^.

**Figure 4:**
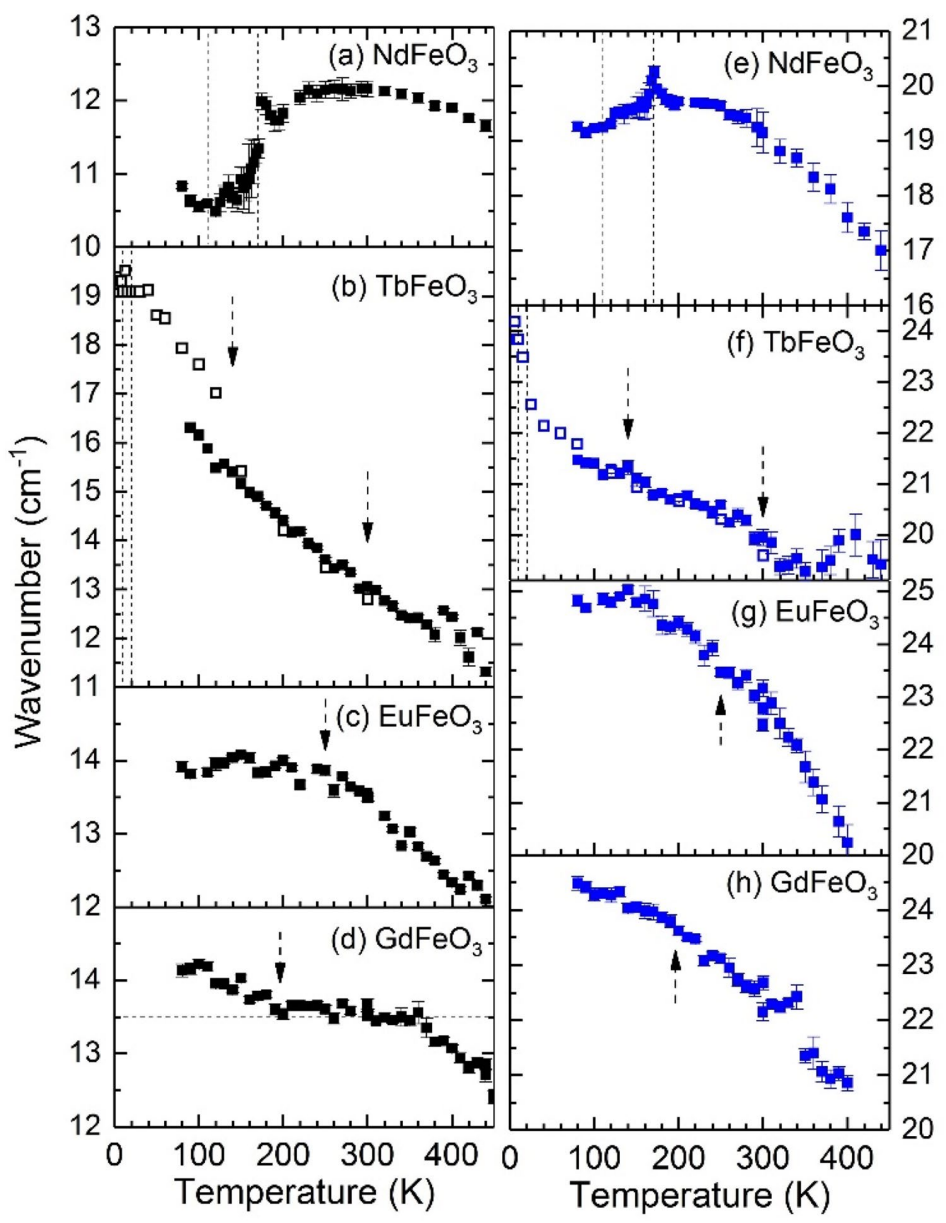
Temperature dependence of the wavenumber of the magnons M1 (left) and M2 (right) observed in NdFeO_3_, TbFeO_3_, EuFeO_3_ and GdFeO_3_. Open symbols for TbFeO_3_ were taken from Ref.^27^. Vertical dashed lines mark the spin reorientation temperatures following Ref.^13^, while dashed arrows mark relevant M(T) anomalies observed in Fig. 1a–d.

Figure 4a–h show the temperature dependence of the wavenumber of the M1 and M2 magnons of NdFeO_3_, TbFeO_3_, EuFeO_3_ and GdFeO_3_, respectively, from 80 to 450 K. For completion, we also show in Fig. 4b,f the available data from the literature between 6 and 300 K for TbFeO_3_. We begin by discussing NdFeO_3_, shown in Fig. 4a,e, which shows a particularly interesting behavior with several regimes. Between 500 and 240 K, the wavenumber of the magnon M1 increases monotonously with decreasing temperature. From 240 to 100 K, the magnon M1 softens by 1.5 cm^−1^. In addition, cusp-like anomalies mark the limits of the spin-reorientation temperature range (170–110 K). Below 100 K, the wavenumber of magnon M1 increases upon further cooling. Following the interpretation given for TbFeO_3_ and SmFeO_3_^26,27^, this result is associated with the alignment of the Nd^3+^ spins in the exchange field of the Fe^3+^ spin sublattice. Qualitatively, our temperature dependence of M1 wavenumber for NdFeO_3_ is in good agreement with the one observed through THz spectroscopy data^30,31^. Magnon M2 hardens on cooling down to 240 K. At the high temperature limit of the spin-reorientation a cusp-like anomaly occurs and, on further cooling down to 78 K, magnon M2 slightly softens.

The temperature dependence of the M1 and M2 magnons wavenumber of TbFeO_3_ here reported (Fig. 4b,f closed symbols) are in good agreement with the already reported data (open symbols), with only the exception of the points between 80 and 100 K, for the case of M1^27^. The wavenumber of both magnons increases monotonously as the temperature decreases. A clear change of slope of the temperature dependence of M1 is observed at around 150 K, in the temperature interval for which the change of slope of the $F(t)$ function is observed (cf. Fig. 1b). The wavenumber of the magnon M2 experiences a sudden increase below 20 K, associated with the spin reorientation transition.

The wavenumber of both magnons of EuFeO_3_, shown in Fig. 4c,g, increases as temperature decreases and no clear anomalous temperature dependence is found down to 80 K. The temperature dependence of the magnon wavenumbers of GdFeO_3_ are depicted in Fig. 4d,h. The wavenumber of the magnon M2 increases monotonously as temperature decreases towards 80 K, without any hint of anomalous behavior. However, the wavenumber of M1 increases as temperature decreases from 450 K down to 350 K, then it becomes temperature independent down to 200 K, and on further cooling, the wavenumber of magnon M1 increases linearly down to 100 K.

Among the studied compounds, the magnon wavenumbers of TbFeO_3_ exhibit the largest temperature variation, reaching more than 6 cm^−1^ for M1, while the smallest variations are found in EuFeO_3_ and are less than 1 cm^−1^ for M1 and 4 cm^−1^ for the M2 mode, in agreement with their respective largest and smallest magnetization changes (cf. Fig. 1). We can conclude that the anomalies identified in the magnetization curves appear to affect only the M1 mode (seen in TbFeO_3_ and GdFeO_3_), while they apparently do not affect the M2 mode, which only presents anomalies at the spin-reorientations (seen in NdFeO_3_ and TbFeO_3_). Having in mind the ferromagnetic origin of the M1 magnons, this result points out that the observed magnetic anomalies in TbFeO_3_ and GdFeO_3_ might be associated with changes of the ferromagnetic interactions.

Comparing the temperature behavior of the magnons wavenumber in NdFeO_3_ to other compounds with spin-reorientation, we conclude that M1 exhibits a similar incomplete softening to those reported for ErFeO_3_, TmFeO_3_ and SmFeO_3_, which has been attributed to the coupling between the Fe^3+^ spins and the *R*^3+^ electronic states^30–33^. In this regard, it seems that M1 has a common temperature dependence for all *R*FeO_3_ with spin-reorientation. In contrast, the published results do not exhibit the softening of M2 neither any anomalies seen at the spin-reorientation limits in both magnons wavenumber^26,27^, which deserves further more detailed studies of spin excitations across the spin-reorientations of these compounds.

### Spin–phonon coupling

**Figure 5:**
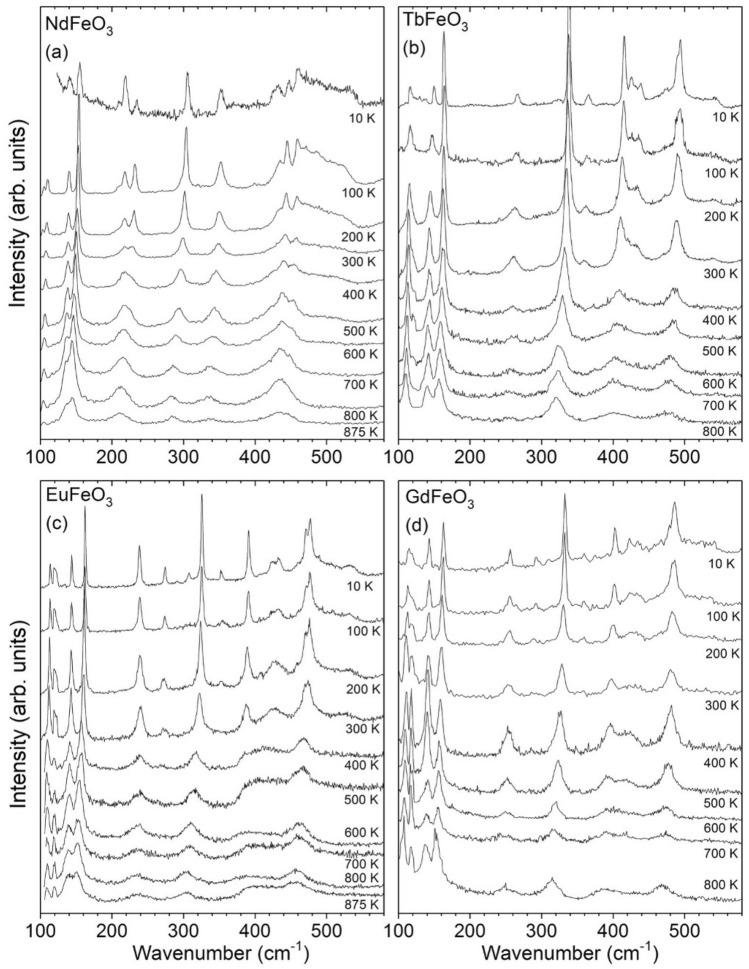
Representative unpolarized Raman spectra of (**a**) NdFeO_3_, (**b**) TbFeO_3_, (**c**) EuFeO_3_ and (**d**) GdFeO_3_, recorded at different fixed temperatures.

In the next step, we investigate the response of the phonon spectra as a function of temperature. The optical phonons of the *R*FeO_3_ have *A*_*g*_, *B*_*1g*_, *B*_*2g*_ and *B*_*3g*_ symmetries—the mode assignment is available in the literature^34^. Figures 5a–d show unpolarized Raman spectra in the range from 100 to 580 cm^−1^, for NdFeO_3_, TbFeO_3_, EuFeO_3_ and GdFeO_3_, respectively, measured at fixed temperatures between 10 and 800 K. The room-temperature Raman signature agrees with literature data^34^. With decreasing temperature, the phonon bands sharpen and shift at different rates. As a consequence, some bands become better resolved and visible.

**Figure 6:**
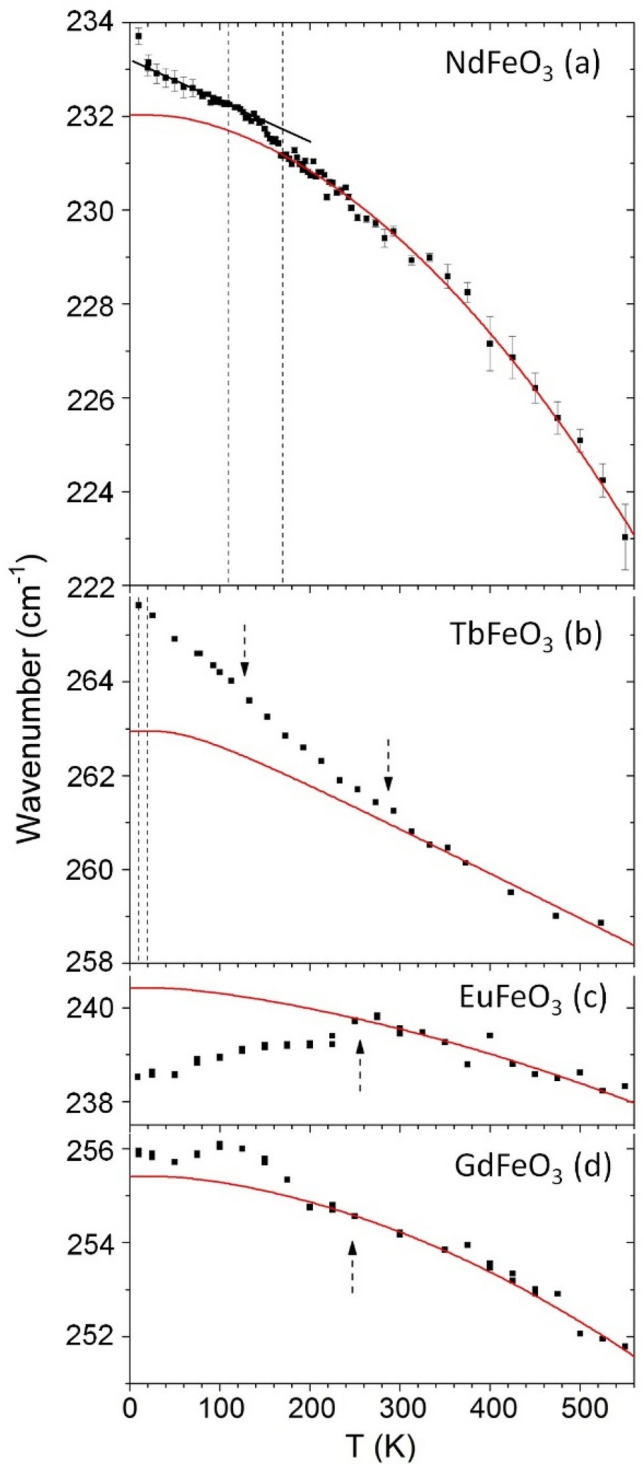
Temperature dependence of the [010]_pc_ in-phase octahedra wavenumber for NdFeO_3_, TbFeO_3_, EuFeO_3_ and GdFeO_3_. The solid curves were determined by the best fits of the anharmonic temperature law above 200 K (for NdFeO_3_ and GdFeO_3_) and 300 K (for TbFeO_3_ and EuFeO_3_) and its extrapolation down to 0 K^35^. Vertical dashed lines mark the phase transition temperatures following Ref.^13^, while dashed arrows mark relevant M(T) anomalies.

**Figure 7:**
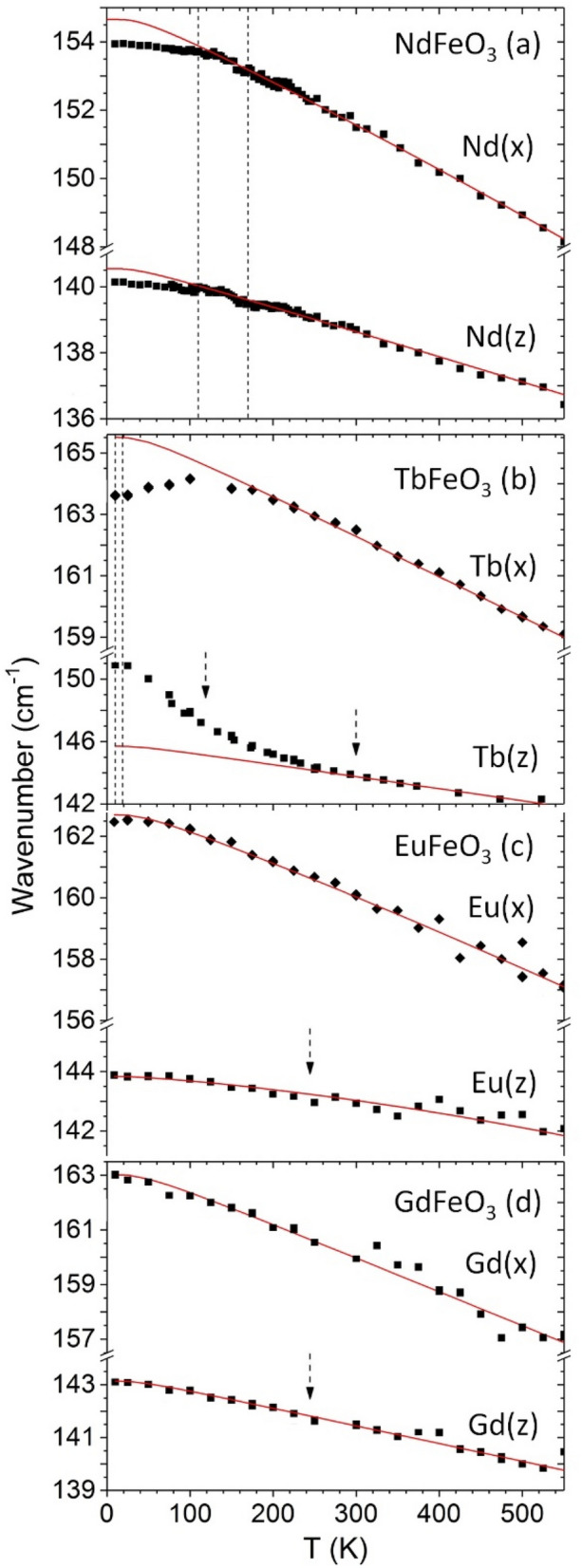
Temperature dependence of the out-of-phase R(x) and R(z)-oscillations wavenumber for NdFeO_3_, TbFeO_3_, EuFeO_3_ and GdFeO_3_. The solid curves were determined by the best fits of the anharmonic temperature law above 200 K (for NdFeO_3_ and GdFeO_3_) and 300 K (for TbFeO_3_ and EuFeO_3_) and its extrapolation down to 0 K^35^. Vertical dashed lines mark the phase transition temperatures following Ref.^13^, while dashed arrows mark relevant M(T) anomalies.

From the study of the temperature evolution of each observed mode, we chose to present and discuss in more detail two modes, which present the most relevant spin–phonon coupling: the [010]_pc_ in-phase FeO_6_ octahedra rotation mode (Fig. 6a–d), and of the out-of-phase *R*-oscillations modes along the *x*- and *z*-axes (Fig. 7a–d). As the magnetic super-exchanges in the Fe^3+^ spin sublattice is tightly associated with the Fe–O–Fe bond angle, the FeO_6_ rotation modes are highly sensitive to magnetic changes of the Fe^3+^ spin sublattice. Moreover, since the *R*-oscillation modes are sensitive to the environment around the R^3+^ cations, we use them as probes of the interaction R–Fe spins interaction. To study the spin–phonon coupling, the phonon wavenumber is compared to the expected anharmonic temperature behavior, obtained from the best fit to the experimental data, above 200 K (for NdFeO_3_ and GdFeO_3_) and 300 K (for TbFeO_3_ and EuFeO_3_), of the equation^35^

2 $$\omega \left( T \right) = \omega _{0} - C\left( {1 + \frac{2}{{e^{{\hbar \omega _{0} /2k_{B} T}} - 1}}} \right) - D\left( {1 + \frac{3}{{e^{{\hbar \omega _{0} /3k_{B} T}} - 1}} + \frac{3}{{(e^{{\hbar \omega _{0} /2k_{B} T}} - 1)^{2} }}} \right),$$

where ω_0_, C and D are fitting parameters and k_B_ is the Boltzmann constant. These fits are given as solid lines in Figs. 6 and 7. It is at first sight surprising that no anomalous temperature dependence is observed at T_N_ (see Fig. S3 of Supplemental Material), also not found for YFeO_3_^36^.

In the [010]_pc_ in-phase FeO_6_ octahedra rotation mode, the spin-reorientation for NdFeO_3_ reveals itself through anomalies in the temperature dependence of this mode observed in Fig. 6a. Its temperature dependence presents a deviation to higher wavenumbers below 170 K. From 150 to 30 K, the wavenumber linearly increases with decreasing temperature. No anomalous temperature dependence at the low temperature limit of the spin-reorientation transition is observed. Below 20 K, a sudden increase of the wavenumber is observed on approaching the compensation temperature of NdFeO_3_ at 8 K. In TbFeO_3_ (Fig. 6b), a strong deviation to higher wavenumbers of the octahedra rotation mode occurs below 300 K. In the case of EuFeO_3_ (Fig. 6c), a downshift takes place below 250 K, while for GdFeO_3_ (Fig. 6d), an upshift is observed below 200 K.

For all the studied compounds, the anomalous temperature dependence of the [010]_pc_ in-phase FeO_6_ octahedra rotation mode wavenumber is observed at the same temperature where the corresponding M(T) curves exhibit anomalies (marked by the dashed lines and arrows in Fig. 6a–d). This implies the specific role of this mode in the spin–phonon coupling of the studied compounds, even at the spin-reorientation of NdFeO_3_. The shifts ranging from 1 to 3 cm^−1^, with the expected magnitude for spin–phonon coupling effects^37–40^, would correspond to octahedra rotation changes, estimated to be between 0.05° and 0.15°, arising from the magnetostructural coupling. The Raman mode wavenumber shift are positive, except for EuFeO_3_. This is associated with the overall different magnetization response of EuFeO_3_ in comparison to the other compounds as shown in Fig. 1, due to the lack of *R*–Fe interactions, which leads to a different spin–phonon interaction. This assumption is actually corroborated by the substantially smaller magnitude of the mean interaction parameter $\langle d\rangle$ of Eu–Fe sublattices relatively to the remaining compounds, as it can be straight confirmed from Table 1.

We now aim at understanding the cross-talk of the magnetic *R*–Fe interaction with the phonon lattice, as recently observed in SmFeO_3_^40^. To do so, we address the out-of-phase *R*-oscillations modes, shown in Fig. 7a–d. In general, three different temperature behaviors of these modes are observed.

Concerning the two Nd-oscillation modes (Fig. 7a), their wavenumbers follow the anharmonic temperature law down to 110 K, below which a small downshift occurs. On further cooling, they both increases linearly, with a maximum downshift of around 0.5 cm^−1^ at 10 K. Although small, this points out for a coupling between the spin structure and the phonons involving the Nd^3+^ cations.

Figure 7b shows the temperature dependence of the wavenumber of the two Tb-oscillation modes. Below 280 K/150 K, the wavenumber of the Tb(z)/Tb(x) oscillation mode shows a notable upshift/downshift, with a maximum magnitude of 4 cm^−1^. Each mode starts its deviation at a temperature wherein an anomaly was found in the M(T) curve, evidencing the interaction of the ordered Fe^3+^ spins with the paramagnetic Tb^3+^ spins. Moreover, the different temperature of deviation and direction of the Tb-oscillation modes wavenumber along each crystallographic axis are evidence of a strong magnetic anisotropy which affects the elastic interactions involving the Tb^3+^ cations.

Finally, for EuFeO_3_ and GdFeO_3_, the wavenumber of both *R*(z) and *R*(x)-oscillation modes follow the expected anharmonic temperature dependence in the investigated range, as shown in Fig. 7c,d, respectively. The absence of a coupling between spins and the *R*-oscillation modes can be understood by the properties of the Eu^3+^ and Gd^3+^ cations. The van Vleck character of Eu^3+^ cation leads to a small magnitude of the mean interaction parameter $\langle d\rangle$ regarding the magnetic Eu^3+^–Fe^3+^ interactions^24^, which are therefore small. On other hand, Gd^3+^ does not exhibit magnetocrystalline anisotropy and, as it has no orbital angular momentum, the spin–orbit interaction is negligibly small. Consequently, a negligible coupling between Gd^3+^ spin orientation changes and cationic oscillations are expected.

To get further information regarding the coupling between phonons and spins, we investigate the quantitative correlation between the measured magnetization and the contribution Δω = ω_ph_(T) − ω_ph-anhar_(T) to the phonon wavenumber due to changes in the spin structure.

**Figure 8:**
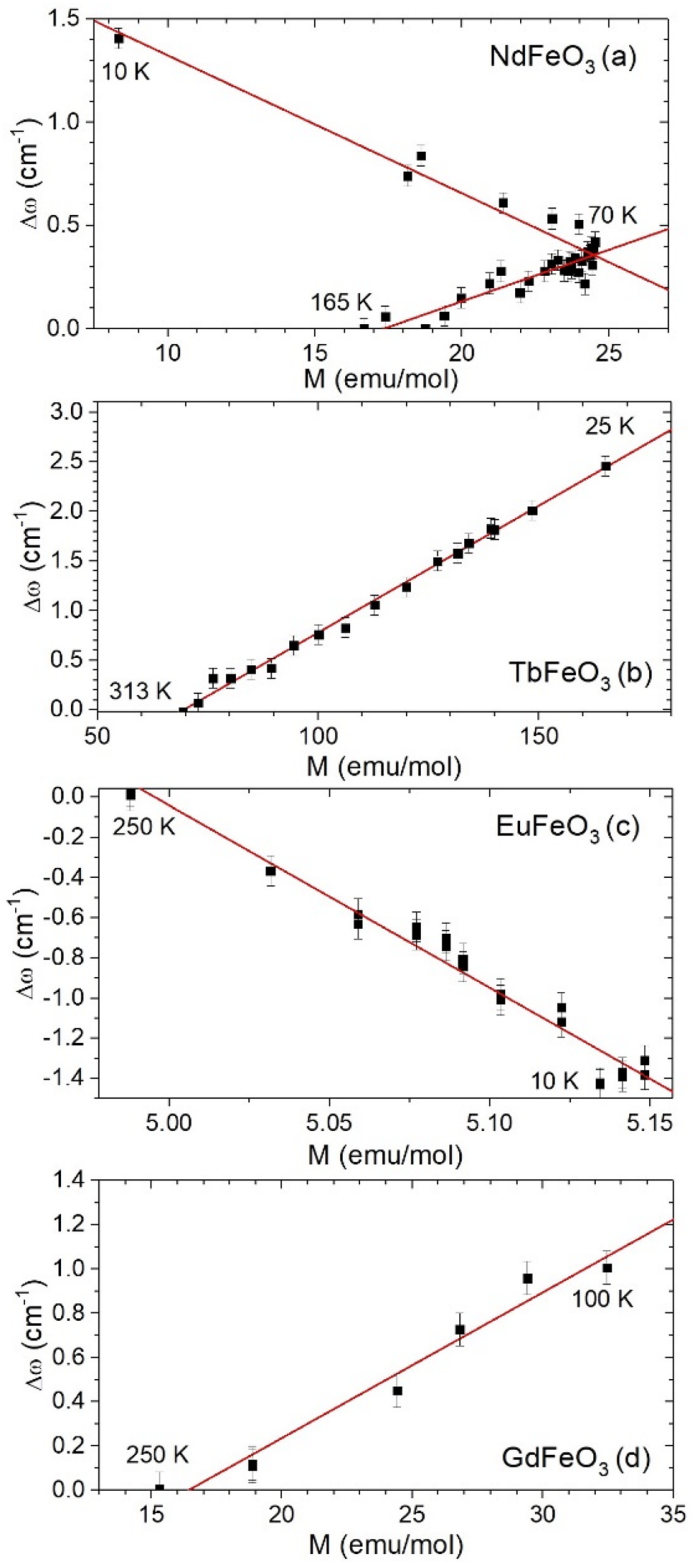
Anomalous contribution to the wavenumber of the [010]_pc_ in-phase octahedra rotations of (**a**) NdFeO_3_ (20–165 K), (**b**) TbFeO_3_ (25–313 K), (**c**) EuFeO_3_ (10–250 K), and (**d**) GdFeO_3_ (100–250 K) as a function of the measured magnetization.

The relation between Δω and the measured magnetization is shown in Fig. 8a–d (see Fig. S4 of Supplemental Material for a wider temperature range). For NdFeO_3_ (Fig. 8a), two linear regimes are found: one below 165 K encompassing the spin reorientation process, and another below 70 K, during the Nd^3+^ spins ordering process. Apparently, the different slopes of the linear relations result from the different mechanisms involved in each process.

For TbFeO_3_, a unique linear dependence is observed, from 313 to 25 K. Below 25 K no linearity is observed, which could be associated with precursor effects of the spin-reorientation taking place at 20 K. In EuFeO_3_ and GdFeO_3_ (Fig. 8c,d), the unique linear dependence found below 250 K shows that these wavenumbers deviations origin from the magnetic changes, seen here via the spin–phonon coupling. In GdFeO_3_ this linearity is lost below 100 K, where strong non-monotonous variations of the M(T) curve are observed. This quantitative analysis allows us to state that the found magnetostructural effect results from a linear spin–phonon coupling that mediates the [010]_pc_ in-phase octahedra rotational phonon and the magnetic structure.

**Figure 9:**
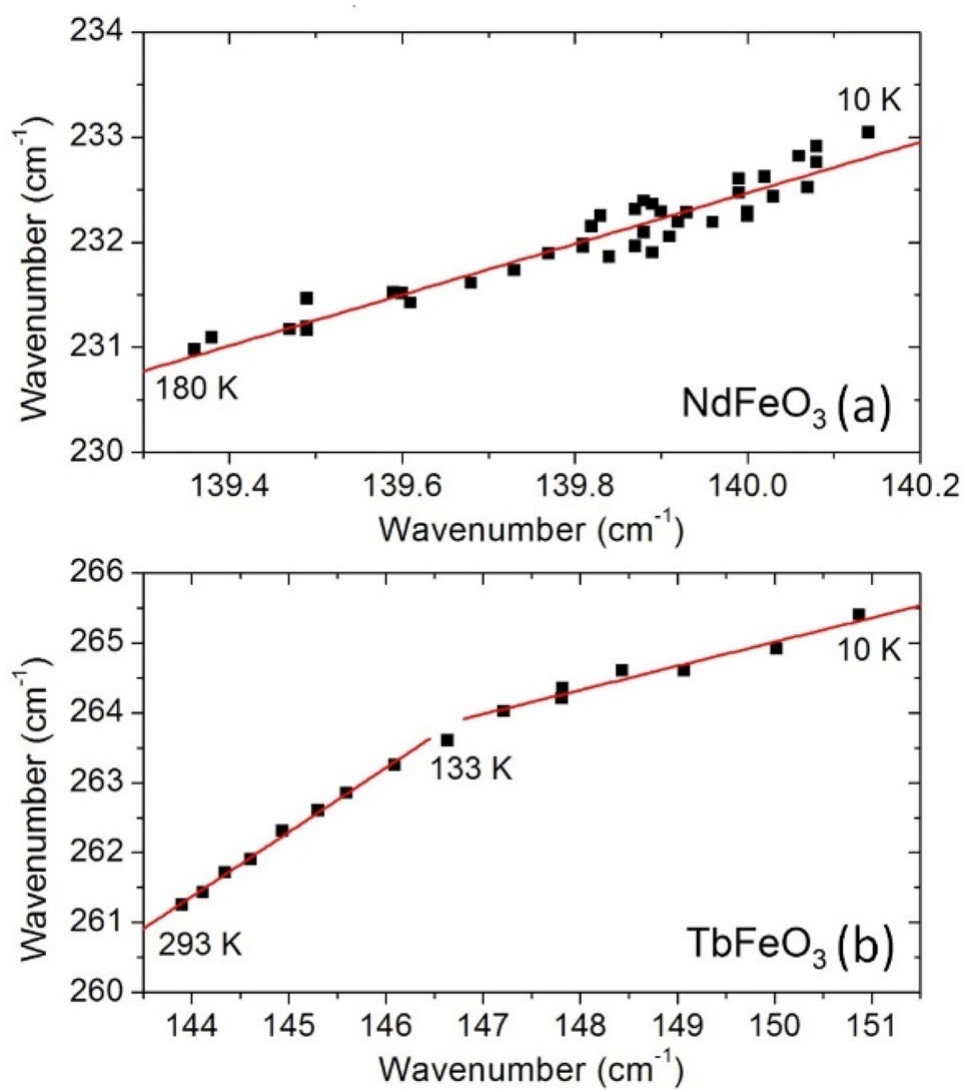
Wavenumber of the [010]_pc_ in-phase octahedra rotation mode as a function of the wavenumber for the (**a**) Nd(*z*) and (**b**) Tb(*z*) out-of-phase oscillation modes, from 10 to 180 K and to 293 K, respectively.

To further evidence that the Nd(z)- and Tb(z)-oscillation modes are sensing the same phenomena of the [010]_pc_ in-phase octahedra rotation mode, we have studied the correlation between their wavenumbers, shown in Fig. 9. We chose the R(z) instead of the R(x), because for the case of TbFeO_3_ only the former shows a deviation similar to the FeO_6_ rotation mode. The linear correlations between their wavenumbers found in the analyzed temperature ranges evidence for a coupling between them, with an unknown phenomenologically proportionality constant. For TbFeO_3_, around 133 K, within the temperature interval where the slope of the linear temperature dependencies of both the $F(t)$ and M1 wavenumber curves changes the most, there is also a change of the found phenomenologically proportionality constant between these modes. This could not be observed in GdFeO_3_ due to the absence of coupling between the Gd^3+^ motions and spins.

## Conclusions

We have reported an experimental study of the magnetization, Raman-active spin excitations and lattice dynamics in *R*FeO_3_ (*R* = Nd, Eu, Gd, and Tb) in the 10 to 850 K range. The main outcomes are summarized in the following.

Besides the known spin-reorientation of NdFeO_3_, anomalies in the temperature dependence of magnetization and wavenumber of both the magnons and the FeO_6_ rotation modes evidence for magnetic changes in TbFeO_3_, EuFeO_3_ and GdFeO_3_, between 25 and 350 K, which are not associated with symmetry breaking nor magnetic phase transitions. The reinforcement of the ferromagnetic response of TbFeO_3_ and GdFeO_3_ is evidenced by both the increasing of magnetization as temperature decreases, and the anomalous temperature behavior of the ferromagnetic mode (magnon M1) at specific temperatures.

The sensitivity of the optical phonons to the Fe–Fe and *R*–Fe magnetic interactions was evidenced. The linear dependence on the magnetization of the anomalous contribution to the phonon wavenumber, just below the temperature where anomalies in M(T) are observed, distinctly bears the coupling between the [010]_pc_ in-phase FeO_6_ rotation mode and the spin structure, through a linear spin–phonon coupling. Thus, the second main outcome concerns the common magnetostructural coupling, evidenced by FeO_6_ octahedra rotations. Like the FeO_6_ rotational modes, the Raman-active *R*-oscillation modes are sensitive to the *R*–Fe magnetic interactions, provided a strong spin–phonon coupling mediated by spin–orbit coupling. In NdFeO_3_ and TbFeO_3_, wherein spin–orbit coupling of the *R*-cations exists, *R*-oscillations are coupled to the [010]_pc_ in-phase octahedra rotational modes. The experimental results give strong evidence for the cross-talk between Fe^3+^ and Nd^3+^/Tb^3+^ spin sublattice, denoting the third main outcome of this work. The largest variations of the magnon M1 and both studied Raman modes are the largest for TbFeO_3_. Moreover, the smaller spin–phonon coupling strength found for the Nd-oscillations in NdFeO_3_ suggests that the anisotropy of the Nd–Fe interaction plays a more important role on triggering the spin-reorientation at higher temperatures rather than its strength. Contrarily though, no deviation of these two modes could be observed for GdFeO_3_, demonstrating the key role played by the spin–orbit coupling to underlie the interplay between spins and the Gd-oscillation phonon.

## Supplementary Information

Supplementary Information.

## Data Availability

The datasets used and/or analyzed during the current study available from the corresponding author on reasonable request.
