# Author Correction: Magnetostructural coupling in *R*FeO_3_ (*R* = Nd, Tb, Eu and Gd)

**DOI:** 10.1038/s41598-023-28181-3

**Published:** 2023-01-18

**Authors:** R. Vilarinho, M. C. Weber, M. Guennou, A. C. Miranda, C. Dias, P. Tavares, J. Kreisel, A. Almeida, J. Agostinho Moreira

**Affiliations:** 1 IFIMUP, Departamento de Física e Astronomia, Faculdade de Ciências, Universidade do Porto, rua do Campo Alegre s/n, 4169-007 Porto, Portugal; 2 Department of Materials, ETH Zurich, Vladimir-Prelog-Weg 4, 8093 Zurich, Switzerland; 3 Institut des Molécules et Matériaux du Mans, UMR 6283 CNRS, Le Mans Université, 72085 Le Mans, France; 4 Department of Physics and Materials Science, University of Luxembourg, 41 Rue du Brill, 4422 Belvaux, Luxembourg; 5 Centro de Química, Departamento de Química, Universidade de Trás-os-Montes e Alto Douro, 5000-801 Vila Real, Portugal

Correction to: *Scientific Reports* 10.1038/s41598-022-13097-1, published online 11 June 2022

The original version of this Article contained an error in the Acknowledgements section.

“We are deeply acknowledged to J. L. Ribeiro for the fruitful discussion regarding the interpretation of the magnetic data, and P. Bouvier suggestions on the analysis of some specific phonons. We acknowledge funding from NORTE-01-0145-FEDER-022096, UID/NAN/50024/2019 and PTDC/NAN-MAT/28538/2017 projects and R.V. to the grant from PTDC/NAN-MAT/0098/2020.”

now reads:

"We are deeply acknowledged to J. L. Ribeiro for the fruitful discussion regarding the interpretation of the magnetic data, and P. Bouvier suggestions on the analysis of some specific phonons. We acknowledge funding from NORTE-01-0145-FEDER-022096, UIDB/04968/2020 and PTDC/NAN-MAT/28538/2017 projects and R.V. to the grant from PTDC/NAN-MAT/0098/2020."

The original Article has been corrected.

