# Supplementary material for: Magnetostructural coupling in RFeO3 (R = Nd, Tb, Eu and Gd)
Source: Sci Rep. 2022 Jun 11;12:9697. doi: 10.1038/s41598-022-13097-1 (PMC9188583; doi:10.1038/s41598-022-13097-1)
Supplement: Supplementary file 1 — Supplementary Information. [file 41598_2022_13097_MOESM1_ESM.docx]

**Magnetostructural coupling in *R*FeO_3_ (*R* = Nd, Tb, Eu and Gd)**

R. Vilarinho,^1,*^ M.C. Weber,^2,3^ M. Guennou,^4^ A. Miranda,^1^ C. Dias,^1^ P. Tavares,^5^ J. Kreisel,^4^ A. Almeida,^1^ and J. Agostinho Moreira^1^

*^1^IFIMUP, Departamento de Física e Astronomia, Faculdade de Ciências, Universidade do Porto, rua do Campo Alegre s/n, 4169-007 Porto, Portugal.*

*^2^Department of Materials, ETH Zurich, Vladimir-Prelog-Weg 4, 8093 Zurich, Switzerland.*

*^3^Institut des Molécules et Matériaux du Mans, UMR 6283 CNRS, Le Mans Université, Le Mans 72085, France*

*^4^Department of Physics and Materials Science, University of Luxembourg, 41 Rue du Brill, L-4422 Belvaux, Luxembourg*

*^5^Centro de Química, Departamento de Química, Universidade de Trás-os-Montes e Alto Douro, 5000–801 Vila Real, Portugal*

*Corresponding author: [rvsilva@fc.up.pt](mailto:rvsilva@fc.up.pt)


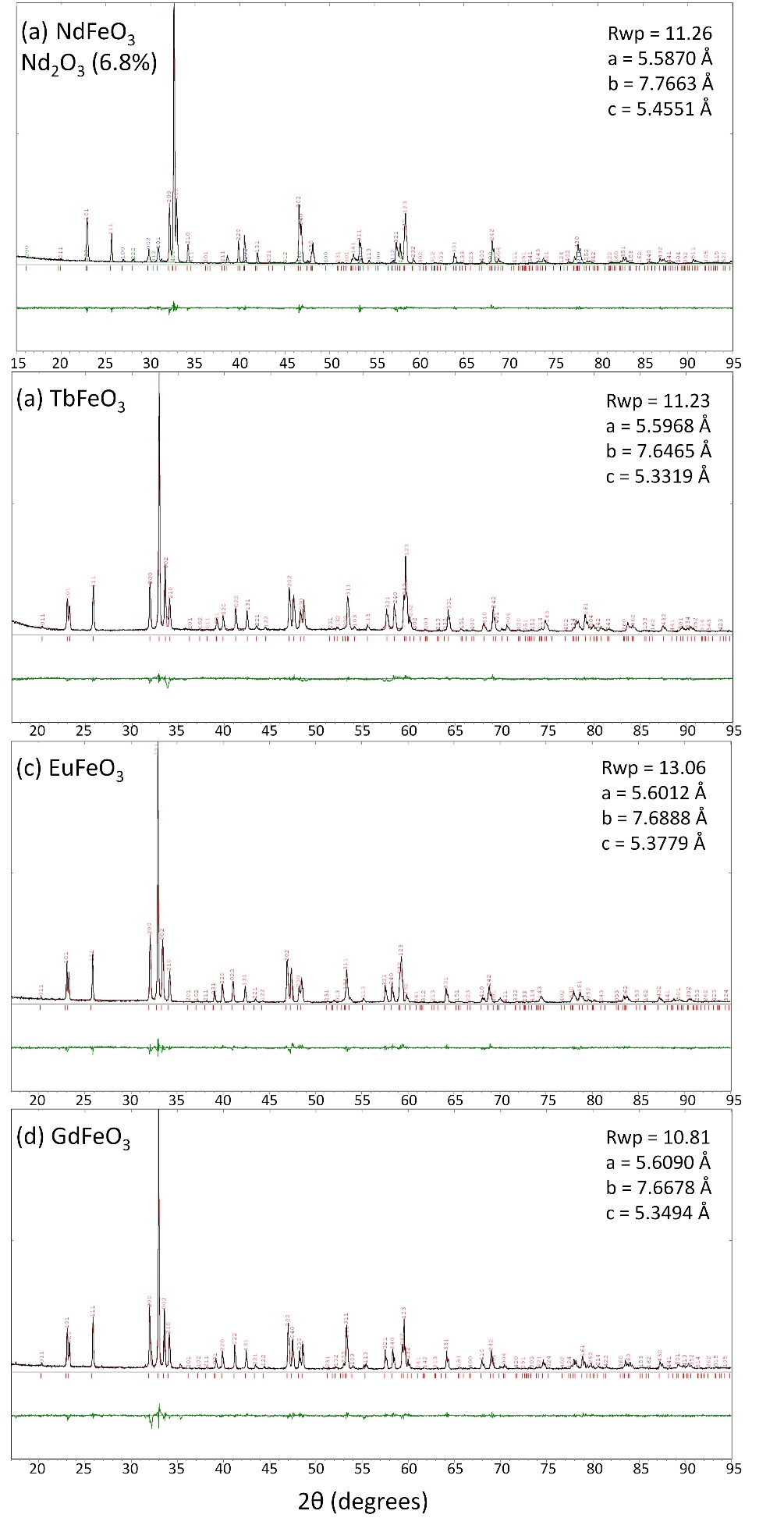


**Figure S1.** Rietveld refinements of the diffraction patterns confirming the *Pnma* space group for (a) NdFeO_3_, (b) TbFeO_3_, (c) EuFeO_3_, and (d) GdFeO_3_, along with the corresponding Rwp agreement factors and lattice parameters. For NdFeO_3_ also a 6.8% amount of Nd_2_O_3_ is found, 6.0% hexagonal (blue ticks) and 0.8% cubic (green ticks).


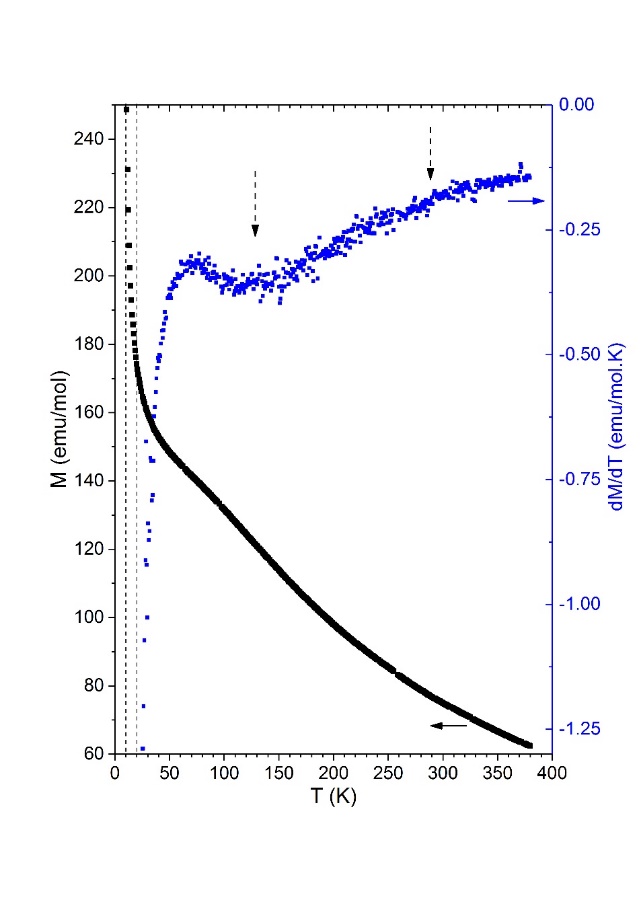


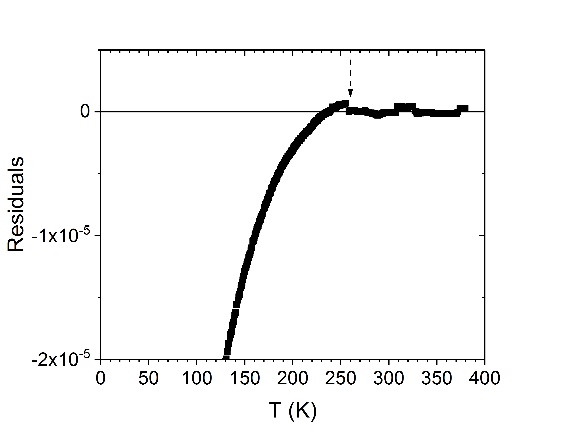


**Figure S2.** Left panel: Detailed view of the magnetization (left axis) curve and its temperature derivative (right axis) for TbFeO3, under ZFC conditions, measured in heating under 40 Oe. Right panel: Temperature dependence of the residuals between M.t/H(T) curve and its best linear fir between 300 and 380 K for TbFeO_3_. Vertical dashed lines mark the phase transition temperatures following Ref. 7, while dashed arrows mark relevant anomalies.


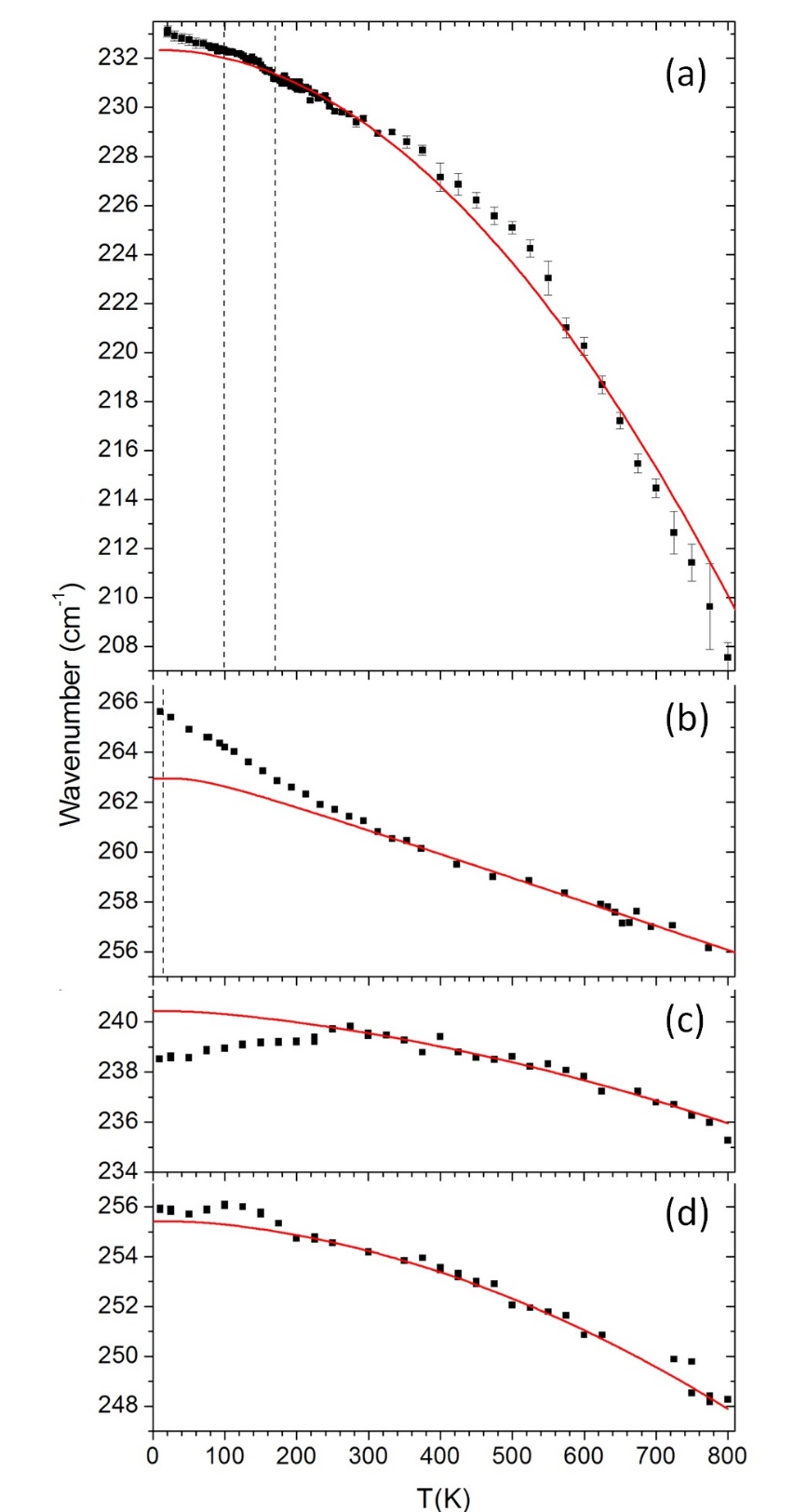


**Figure S3.** Temperature dependence of the wavenumber of the [010]_pc_ in-phase octahedra of (a) NdFeO_3_, (b) TbFeO_3_, (c) EuFeO_3_, and (d) GdFeO_3_. The solid curves were determined by the best fits of Equation (2) above 200 K (for NdFeO_3_ and GdFeO_3_) and 300 K (for TbFeO_3_ and EuFeO_3_), and their extrapolations down to 10 K. Vertical dashed lines the temperature range of the spin-reorientation transition.


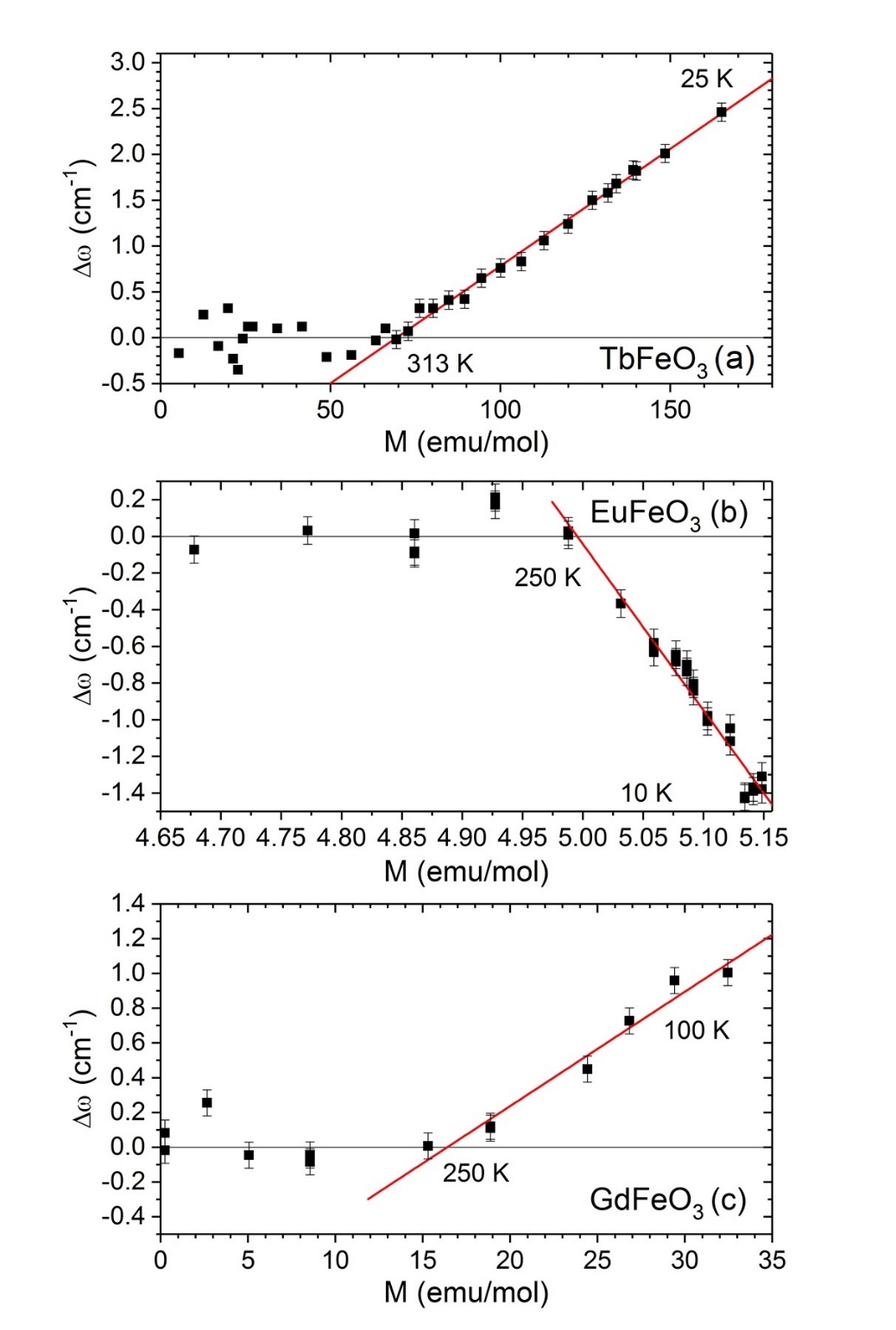


**Figure S4.** Wider temperature range of the anomalous contribution to the wavenumber of the [010]_pc_ in-phase octahedra rotations of (a) TbFeO_3_, (b) EuFeO_3_ and (c) GdFeO_3_ as a function of the measured magnetization.
